# Chronic inflammation: is it the driver or is it paving the road for malignant transformation?

**DOI:** 10.18632/genesandcancer.64

**Published:** 2015-05

**Authors:** Kambiz Afrasiabi, Yi-Hong Zhou, Angela Fleischman

**Affiliations:** ^1^ Department of Medicine, University of California, Irvine, CA, USA; ^2^ Department of Surgery, University of California, Irvine, CA, USA

**Keywords:** Chronic inflammation, Free energy, driver mutation, bioenergetics, metabolomics, electric field and cancer, JAK2^V617F^, p53 Rb

## Abstract

Chronic inflammation in well-defined mouse models such as Giα2 knock out mouse has been shown to trigger formation and expansion of hypoxic niches and also leads to up regulation of NFĸB, offering cells which have adapted their genetic machinery to hypoxia a unique survival advantage. These adapted cells have been shown to acquire stem cell-like capabilities as shown by up regulation of stem cell markers. Such long lived cells become permanent residents in sub mucosa and acquire a malignant phenotype from long-term exposure to noxious environmental agents due to a barrier defect secondary to down regulation of barrier proteins such as Zo1 and Occludin. Indeed mitotic spindle disorientation in such mice has been proposed as another contributory factor to malignant transformation. Sterilization of bowel lumen of these mice through different techniques has prevented malignant transformation in the presence of chronic inflammation. These facts stand strongly against chronic inflammation as a true driver of carcinogenesis but clearly support its role in facilitating the emergence of the neoplastic clone.

## INTRODUCTION

The malignant transformation as per the free energy concept necessitates a significant and permanent decrease in the free energy of the malignant cell as a result of breakdown of the fine balance of cellular energetics[2]. Warburg effect offers a credible reflection of these deeply seated perturbances in cellular energetics and metabolism[4]. If chronic inflammation in well defined and valid mouse models would directly lead to break down of cellular energetics, then the assignment of a driver role to it would become meaningful. However evidence points to the contrary as discussed below. Indeed the cells that evolve in the inflammatory microenvironment under the effect of a myriad of cytokines and chemokines and adapt to the hypoxic niche, however fastidious continue to enjoy normal metabolism with no traces of Warburg effect in them[5].

There are many examples in which chronic inflammation is associated with carcinogenesis. However in none of them there is solid proof of a driver role for chronic inflammation[6,7]. In case of H. Pylori related maltoma, early in the natural history of the disease cure can be obtained by eradicating H. Pylori with antibiotic[8]. Many people with chronic gastritis from H. Pylori never develop malignancy[9], demonstrating that chronic inflammation in and of itself is not sufficient to cause malignant transformation. Epstein Barr virus and its relation with Burkitt's lymphoma in sub-Saharan Africa and nasopharyngeal carcinoma in China provide additional associations between inflammation and malignancy. Although inflammatory cells are observed in the tumor stroma, there is no proof of chronic inflammation as the driver which is best defined as an agent the removal of which would lead to resolution of the malignant phenotype[10].

Viruses can directly induce malignancy by down-regulating tumor suppressor genes such as TP53 and Rb. Through this mechanism papilloma virus promotes the pathogenesis of malignancies such as squamous cell carcinoma of cervix and head and neck[11]. The list of virus associated malignancies is quite long and includes HHV8 with Kaposi sarcoma[12], HIV and a multitude of hematologic and non-hematologic malignancies[13], HTLV1 and adult T cell leukemia and lymphoma endemic in Caribbean and Japan[14], and chronic hepatitis B and C and hepatocellular carcinoma[15].

### Inflammation in hematologic malignancies

There is also evidence that chronic inflammation facilitates clonal evolution in myeloproliferative neoplasm (MPN), a chronic hematologic malignancy. Chronic inflammation is a characteristic feature of MPN and has been implicated as the cause of many of the debilitating symptoms associated with this disease. Emerging evidence suggests that inflammation may play a more central role in disease initiation and maintenance of the neoplastic clone16. Somatic acquisition of a *JAK2^V617F^* mutation in a hematopoietic stem cell is seen in the majority of patients with MPN. *JAK2^V617F^* confers upon hematopoietic progenitors resistance to the suppressive actions of TNF. An environment with high levels of inflammatory cytokines (such as TNF) selects for cells which have mutated in such a way to avoid these suppressive cues (such as the *JAK2^V617F^* neoplastic clone). The *JAK2^V617F^* mutant clone then expands and leads to unrestrained expansion of mature myeloid cells. The *JAK2^V617F^* neoplastic clone in MPN likely also induces an inflammatory state which further enhances its selective advantage. This inflammatory response elicited by the *JAK2^V617F^* clone may also be required for clinical manifestations of the disease. Clones with leukemia-associated mutations (such as *JAK2^V617F^*) can be detected in a few percent of normal aged individuals[17]. This suggests that presence of a clone with a leukemia-associated mutation is not sufficient to induce a clinical hematologic malignancy, and possibly inflammation is necessary to enhance the growth of the clone and lead to clinical disease.

### Inflammation facilitates malignant transformation in glioma

**Figure 1:**
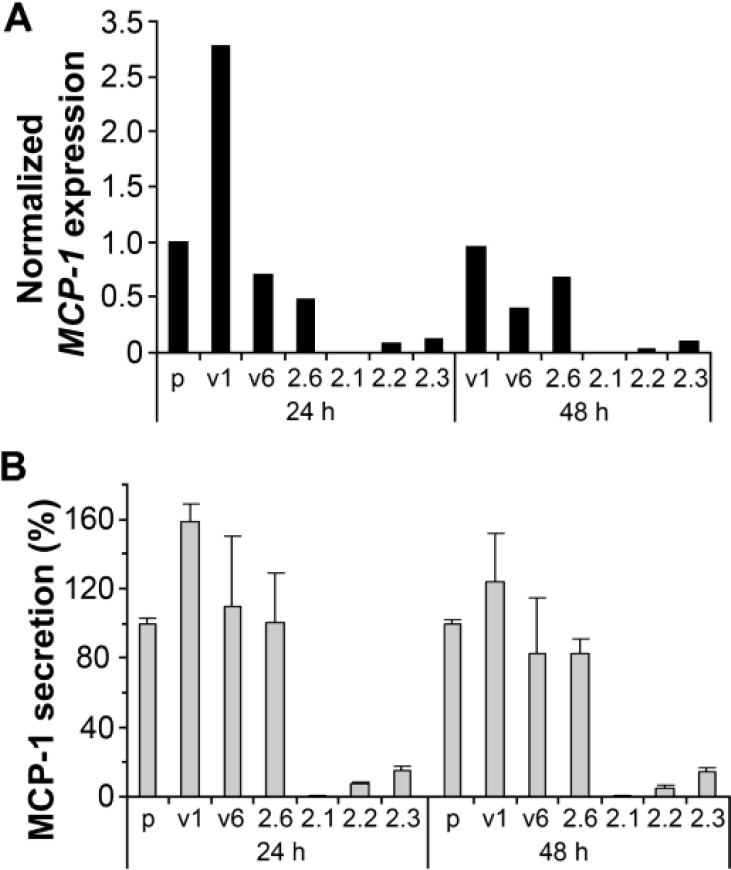
Suppressed expression of CCL2 (MCP-l) in the GBM cell line U251HF expressing transfected PAX6 (2.1, 2.2, and 2.3) as compared with non-transfected (p), vector transfected (vi, v6) and negative PAX6-transfected (2.6) cells Cells were grownin DMEM/F12 under normoxic condition with serum deprivation for 24 and 48 hours before getting subjected to the assays. **(A)** Real-time qRT-PCR measurement of CCL2 expression normalized to ACTB and compared to the level in U25 1HF (p) with serum deprivation for 24 hours, using SYBR-Green I master mix (Roche) and Light Cycler real time PCR instruments following methods described previously 1. **(B)** ELISA quantification of secreted MCP-1 from the same cells analyzed in A, using MCP-1 ELISA kit from Assay Designs (Michigan). Values include the mean + SD of 3 independent experiments. PAX6 level in each cell line / transfectants by western blot assay was shown in ref. 3.

There is evidence supporting inflammation as a tumor growth promoting but not initiating factor. One example is infiltration of glioma associated macrophage/microglia (MG). In CNS inflammation, MG have been shown to be capable of presenting antigens and activating T cells, thus playing a direct role in modulating brain inflammation[18]. A significant number of blood-born monocytes infiltrate into the tumor site[19]. Their trafficking to tumor sites has been proven to promote tumor growth by secreting mitogenic, angiogenic, and immunosuppressive cytokines rather than attacking glioma cells[20,21]. CCL2 (also known as MCP-1), a chemokine that attracts macrophages, is consistently overexpressed in a number of glioma cell lines and in some human high grade gliomas with MG infiltration[22,23]. PAX6 has been shown to play a tumor suppressor role in glioblastoma[24,25]. CCL2 expression in glioma cells was nearly abolished by overexpression of PAX6 (Figure 1). This demonstrates that in glioma inflammation facilitates malignant transformation.

### Inflammation and free energy

One could arbitrarily categorize malignant disorders into two major groups. The first group would be those malignancies that are driven down an inflammatory path, such as the examples mentioned above. The second group would be those malignant disorders that are driven down the non-inflammatory path, such as certain well defined syndromes including Li-Fraumini and Lynch syndrome, classical lung, breast and colon cancer among many others.

Free energy was first described by the American engineer Willard Gibbs in 1873 as the amount of energy available to do non-volume increasing work in a system with constant temperature and pressure and that inversely correlates with the amount of entropy of that system on thermodynamics arrow of time.

Biological systems including a living cell are well known for their unique capability to keep entropy at the lowest possible level dictated by the limits of the second law of thermodynamics. The breakdown of this fine balance which is the distinguishing hallmark of malignant disorders could be envisioned as the universal driving force capable of driving down both inflammatory and non-inflammatory roads of malignant transformation. The fine balance of the cellular energetics under normal condition is governing a broad spectrum of fundamental biological events extending from epigenome to Micro RNA network and incorporating dynamics of mitosis and homeostasis of cell cycle kinetics to DNA damage response and gene regulatory mechanisms[26,27]. Even though we might all be taken by surprise, chronic inflammation might simply be a reaction against low free energy cells and proteins and this could revolutionize our understanding of the pathogenesis of a broad spectrum of as yet puzzling disorders ranging from autoimmune and collagen vascular disorders to cancer. Clearly this represents a different perspective of the reality that we have become used to.

Interestingly enough even the contemporary literature has started to appreciate the interplay of metabolomics and inflammation[28]. Literature has not as yet looked at the potential role of the immune system in recognizing cells and proteins with lower free energy. Rather, the immune system has thus far been looked at as a system comprised of cells and humoral factors specialized in recognizing and destroying foreign cells and antigens as well as microorganisms. Even though HLA system and its biology have acted as the cornerstone of recognizing self and non-self, the sheer discrimination between self and non-self has not been given any deeper and more fundamental attribute. This difference might simply be based on the difference between the free energy of these cells and proteins and self-cells and proteins. Most recently we have been witnessing a new wave of immune mediated strategies taking advantage of checkpoint inhibitors such as PD-1 and CTLA 4 antagonists in unleashing immune system against cancer[29]. Ex vivo training and modification of immune cells has also led to new treatment modalities such as provenge in metastatic prostate adenocarcinoma and CART in the treatment of acute leukemia[30,31]. Even though there is no cure in sight so far, the fact that unleashing the immune system could lead to eradication of malignant cells much more than the normal host cells which are totally different in their free energy supports this notion.

According to the universal free energy concept normal inflammatory cells with normal metabolism and energetics get attracted to the tumor sites comprised of a significant number of cells with significantly lower free energy/lower in situ vibratory motion[2]. This attraction could be envisioned as an attempt at increasing regional free energy by destroying low free energy cancer cells. Many times cancer cells are equipped with the necessary means to disable the inflammatory cells. This inability of immune system leads to further augmentation of the inflammatory response inside the tumor microenvironment which is also responsible for some of the devastating constitutional symptoms that the cancer patient experiences. Indeed a significant number of solid malignancies station in regional lymph nodes and take advantage of the humoral survival and growth promoting factors inside the lymph node microenvironment before spreading hematogenously to other sites. However therapeutic recruitment of sophisticated immune cells to tumor sites which have significantly lower free energy could dramatically facilitate our future treatment strategies in treatment of cancer. For example if we could deliver nanotubes with the desired vibratory potential/jiggling loaded on immune cells which could dock on cancer cells we might come with some breakthroughs in our immune mediated cancer therapeutics. Rejection of the transplanted organs could also be prevented through similar refinements. Finally some of the famous misunderstood drivers of malignant path such as RAS in adenocarcinoma of pancreas, HER family in breast cancer, EGFR in lung cancer and gliomas, all have one thing in common, namely increasing the pace of mitosis of low free energy cancer cells, a futile attempt at increasing the free energy of cancer cells which could potentially lead to inflammatory response in the tumor microenvironment and promote debilitating cancer associated symptomatologies such as fatigue and weight loss.

## CONCLUSION

Although chronic inflammation has been noticed and reported for a long time by many different observers both in the vicinity of malignant cells and at the premalignant lesions of a wide range of malignant disorders both of hematological and solid subtypes, assigning a driver role to chronic inflammation has faced insurmountable technical as well as evidence based barriers.

CCL2 expression on the surface of glioma cells which attracts macrophages to the tumor site, with its ensuing activation of other components of immune system through secretion of humoral factors on one end and eradication of inflammation without any observable effect on tumor growth and progression on the other end, are among the examples that take away the designation of driver role from chronic inflammation in inflammation associated malignancies.

Available evidence supports a promoting role for chronic inflammation following malignant transformation. Interestingly enough at times the malignant cell itself is the source of chemokines which attract the inflammatory cell to tumor site as mentioned above. Finally In other tumor types the malignant cells take advantage of the nurturing microenvironment of the immune system organ, namely lymph nodes before progressing into advanced stages. Melanoma and breast carcinoma are among such malignancies.

The modern generation of immunotherapy, including CTLA4 and PD-1 antagonists, has enabled us to make clinical achievements and prolong the life of patients with metastatic melanoma.

However the toxicities associated with these treatment modalities which originate in reacting against the self-antigens at times have proven prohibitive. The key question is if the immune system can recognize the difference between the energetics of the tumor cell and normal cell, why is it so incapable of destroying tumor cells and in addition why does it contribute to promotion and progression of malignant phenotype?

The answer to this question might reside in the exceptional capability of the tumor cell to also change the energetics of the infiltrating immune cells in favor of a low free energy state which would disable these cells as a result of modification of the quaternary structure of its constituent functional proteins under low free energy condition, in addition to taking advantage of their growth promoting humoral factors. A new wave of improved understanding and design of delicate measurement devices of cellular energetics and the ability to reverse those differences offers hope for future cancer therapeutics modalities. Indeed even today we are witnessing a crude, however rewarding attempt at physical rather than chemical cancer therapy by alternating electrical fields in the treatment of recurrent Glioblastoma multiforme, called NovoTTF-100A system which has been misunderstood as only a change in electrical field of cancer cell without appreciating the induced transient increase in free energy of the cancer cell[32,33].
